# Editorial: Experimental and computational aspects of bioactive proteins from animal venoms: an insight into pharmacological properties and drug discovery

**DOI:** 10.3389/fphar.2024.1380193

**Published:** 2024-02-16

**Authors:** Michelle Khai Khun Yap, Cassandra M. Modahl, Steven R. Hall

**Affiliations:** ^1^ School of Science, Monash University Malaysia, Bandar Sunway, Malaysia; ^2^ Centre for Snakebite Research and Interventions, Liverpool School of Tropical Medicine, Liverpool, United Kingdom; ^3^ Lancaster Medical School and Biomedical and Life Sciences, Lancaster University, Lancaster, United Kingdom

**Keywords:** animal toxins, therapeutics, bioinformatics, omics, molecular pharmacology

The fascinating realm of animal venoms is a treasure trove of bioactive proteins, known as toxins, that are responsible for life-threatening pathological conditions in envenoming. Despite their deadly nature, they have promising potential to be developed into life-saving drugs. For example, an angiotensin-converting enzyme (ACE) inhibitor, captopril, from *Bothrops jararaca* venom has been developed for the treatment of life-threatening hypertension (Smith and Vane, 2003). To harness this potential, a comprehensive understanding of animal venom toxins (e.g., intricating their structures, functions, and mechanistic actions) is essential for therapeutic discovery and antivenom development (Kini, 2010). Recent advancements in biotechnologies have facilitated deeper insights into animal venoms. The use of omics (e.g., genomics, transcriptomics and proteomics) in venom research, termed venomics, seeks to describe the diversity and complexity of venoms, providing the foundation for toxin characterisation, and antivenom development (Modahl et al., 2020; Tan, 2022). These approaches characterise the pharmacological properties of toxins through the convergence of experimental and computational methods. Indispensable computational studies of toxins encompass molecular modelling, docking and simulations of molecular dynamics delineate structure-function relationships, especially interactions with cellular targets that drive pharmacological effects (Ojeda et al., 2017).

Our Research Topic collected 9 articles of new findings and perspectives to unravel the pharmacological and drug discovery potentials of venoms from spiders, snakes and bees.

Understanding the mechanistic actions of toxins on molecular targets is the first step towards the development of novel therapeutics. Li et al. expressed a novel toxin, LCTX-F2, identified from spider *Lycosa singoriensis* venom, and performed a series of characterisation experiments. LCTX-F2 is a 7.5 kDa peptide with 65 amino acids and belongs to the toxin 35 superfamily in spider venoms. Notably, this toxin possesses procoagulant activity with significant reduction in clotting times through interaction with coagulation factors FXIIa, Fxa, kallikrein, and thrombin. This study demonstrated a dose-dependent decrease in bleeding time in experimental mouse models with no cytotoxic or haemolytic activities. These findings suggest the potential of LCTX-F2 as a future procoagulant drug.

Badari et al. discovered a cysteine-rich secreted protein (CRISP) isoform they have named patagonin in the venom of a snake, *Philodryas patagoniensis*, by reverse-phase HPLC bioassay-guided fractionation. Patagonin exhibited significant antimicrobial effects against *Pseudomonas aeruginosa* and *Penicillium expansum*. Patagonin is also non-cytotoxic and non-haemolytic, highlighting snake venoms as promising sources of novel antimicrobial molecules.

Chan et al. discovered ZK002, a 30 kDa protein from *Deinagkistrodon acutus* venom with anti-angiogenic and anti-inflammatory effects. This protein targets VEGF signalling pathways and angiogenic-related proteins. It also suppresses pro-inflammatory cytokines which demonstrates the potential of ZK002 as dual-functional therapeutic agents for angiogenesis and inflammation-related diseases.

The scorpion venom toxin has also been found to exhibit anticancer properties. In a study by Mlayah-Bellalouna et al., P01 toxin from *Androctonus australis* possesses selective anticancer effects against glioblastoma cells, by targeting SK2 channels. Their findings proposed the potential therapeutic development of P01 for glioblastoma.

Shi et al. reviewed the pharmacological potentials of bee venoms. This review covers a comprehensive appraisal of the pharmacological effects of bee venoms and their principal components responsible for their anticancer, anti-inflammatory, anti-infective, analgesic and neuroprotective effects. The authors applied bioinformatic analyses, KEGG pathway analyses, and protein-protein interaction (PPI) analyses to predict mechanistic actions and signalling pathways which represent important molecular pharmacology of bee venoms. The review also provides a thorough overview of potential therapeutic applications of bee venoms through network pharmacology.

In addition to characterising toxin activities, it is of great importance to understand the structure-function relationship of toxins and their molecular targets. Dongol et al. investigated structure-function relationships of a spider venom toxin, Ssp1a, a selective sodium channel (Nav) subtype antagonist. They adopted molecular docking to design two promising Ssp1a analogues, S7R-E18K-rSsp1a and N14D-P27R-rSsp1, with enhanced potency and selectivity for NaV subtypes.

From here, they successfully developed S7R-E18K-rSsp1a and N14D-P27R-rSsp1, two promising analogues with improved efficacy and selectivity towards NaV subtypes, NaV1.3 and NaV1.2/1.7, respectively. These findings emphasise how computational approaches could be applied in rational drug design from toxins to achieve therapeutic outcomes for medical conditions associated with sodium channel subtypes.

Although we observe great therapeutic potentials in certain animal venom toxins, we also appreciate that it is the diversity of the animal venom toxins which can make the treatment of envenoming challenging. One of these challenges is the immunogenicity of venom toxins, which requires study to develop better antivenoms targeting specific toxins (Hiu et al., 2023) and exhibiting broader neutralising potency. In this regard, Chan et al. examined immunogenicity of α-neurotoxins from elapid venoms. Neurotoxins are lethal toxins found predominantly in elapid venoms which cause neuromuscular paralysis in envenomed victims. Current antivenoms exhibited low neutralisation efficacy for neurotoxins. Herein, the authors used a structure-based prediction model analysis and a DM-editing determinant screening algorithm to evaluate the immunogenicity of α-neurotoxins from five Asiatic elapid venoms - *Naja kaouthia*, *Ophiophagus hannah*, *Laticauda colubrina*, *Hydrophis schistosus*, and *Hydrophis curtus*. It was found that the low immunogenicity of neurotoxins could be attributed to the smaller size and amino acid compositions, hindering the effectiveness of existing antivenoms. The authors proposed the use of synthetic epitopes to develop more effective neurotoxin neutralising antivenoms.

Drug repurposing has also been an alternative, accessible and cost-effective treatment option for envenoming (Koh et al., 2020; Puzari et al., 2021; Hall et al., 2023). Herein, Clare et al. developed a high-throughput screening (HTS) panel on repurposed drug libraries and identified 14 repurposed drugs that can antagonise snake venom metalloproteinases (SVMPs), a known toxin family that causes haemorrhage and coagulopathy. In addition, a similar HTS pipeline was applied in parallel for another venom toxin, i.e., phospholipase A_2_ (PLA_2_) as discussed by Albulescu et al. These HTS pipeline methodologies offer automated and rapid strategies to discover drug “hits” from diverse chemical libraries as potential future envenoming therapeutics.

Overall, this Research Topic highlights the comprehensive investigations into experimental and computational facets within animal venom research, paving the way for profound insights and transformative drug discovery. The application of bioinformatics and computational tools enables holistic improvement in the identification of promising leads from animal venoms, forming a paradigm shift in drug discovery. Moving forward, artificial intelligence (AI) integration in animal venom research will likely revolutionise therapeutics discovery in a more streamlined and guided manner. Additionally, database curation of potential hits from animal venoms requires collation of diverse data, aiding in therapeutic development for venom research and promoting interdisciplinary collaboration.

## References

[B1] Hall S. R. Rasmussen S. A. Crittenden E. Dawson C. A. Bartlett K. E. Westhorpe A. P. (2023). Repurposed drugs and their combinations prevent morbidity-inducing dermonecrosis caused by diverse cytotoxic snake venoms. Nat. Commun. 14, 7812. 10.1038/s41467-023-43510 38097534 PMC10721902

[B2] Hiu J. J. Fung J. K. Y. Tan H. S. Yap M. K. K. (2023). Unveiling the functional epitopes of cobra venom cytotoxin by immunoinformatics and epitope-omic analyses. Sci. Rep. 13, 12271. 10.1038/s41598-023-39222-2 37507457 PMC10382524

[B3] Kini R. M. (2010). Toxin treasure in snake venoms: a protein biochemist's sandbox. Biochem. 1 32 (4), 24–28. 10.1042/BIO03204024

[B4] Koh C. Y. Bendre R. Kini R. M. (2020). Repurposed drug to the rescue of snakebite victims. Sci. Transl. Med. 12 (542), eabb6700. 10.1126/scitranslmed.abb6700 32376770

[B5] Modahl C. M. Brahma R. K. Koh C. Y. Shioi N. Kini R. M. (2020). Omics technologies for profiling toxin diversity and evolution in snake venom: impacts on the discovery of therapeutic and diagnostic agents. Annu. Rev. Anim. Biosci. 8, 91–116. 10.1146/annurev-animal-021419-083626 31702940

[B6] Ojeda P. G. Ramírez D. Alzate-Morales J. Caballero J. Kaas Q. González W. (2017). Computational studies of snake venom toxins. Toxins 10 (1), 8. 10.3390/toxins10010008 29271884 PMC5793095

[B7] Puzari U. Fernandes P. A. Mukherjee A. K. (2021). Advances in the therapeutic application of small-molecule inhibitors and repurposed drugs against snakebite. J. Med. Chem. 64 (19), 13938–13979. 10.1021/acs.jmedchem.1c00266 34565143

[B8] Smith C. G. Vane J. R. (2003). Discov. captopril. FASEB J 17 (8), 788–789. 10.1096/fj.03-0093life 12724335

[B9] Tan C. H. (2022). Snake venomics: fundamentals, recent updates, and a look to the next decade. Toxins 14 (4), 247. 10.3390/toxins14040247 35448856 PMC9028316

